# Excluding spatial sampling bias does not eliminate oversplitting in DNA‐based species delimitation analyses

**DOI:** 10.1002/ece3.7836

**Published:** 2021-07-02

**Authors:** Daniel Lukic, Jonas Eberle, Jana Thormann, Carolus Holzschuh, Dirk Ahrens

**Affiliations:** ^1^ Zoologisches Forschungsmuseum Alexander Koenig Zentrum für Taxonomie und Evolutionsforschung Bonn Germany; ^2^ Zoologische Evolutionsbiologie Paris‐Lodron‐Universität Salzburg Austria; ^3^ Villach Austria

**Keywords:** barcoding, *cox1*, geographic sampling bias, Laos, species delimitation

## Abstract

DNA barcoding and DNA‐based species delimitation are major tools in DNA taxonomy. Sampling has been a central debate in this context, because the geographical composition of samples affects the accuracy and performance of DNA barcoding. Performance of complex DNA‐based species delimitation is to be tested under simpler conditions in absence of geographic sampling bias. Here, we present an empirical dataset sampled from a single locality in a Southeast‐Asian biodiversity hotspot (Laos: Phou Pan mountain). We investigate the performance of various species delimitation approaches on a megadiverse assemblage of herbivorous chafer beetles (Coleoptera: Scarabaeidae) to infer whether species delimitation suffers in the same way from exaggerate infraspecific variation despite the lack of geographic genetic variation that led to inconsistencies between entities from DNA‐based and morphology‐based species inference in previous studies. For this purpose, a 658 bp fragment of the mitochondrial cytochrome c oxidase subunit 1 (*cox1*) was analyzed for a total of 186 individuals of 56 morphospecies. Tree‐based and distance‐based species delimitation methods were used. All approaches showed a rather limited match ratio (max. 77%) with morphospecies. Poisson tree process (PTP) and statistical parsimony network analysis (TCS) prevailingly over‐splitted morphospecies, while 3% clustering and Automatic Barcode Gap Discovery (ABGD) also lumped several species into one entity. ABGD revealed the highest congruence between molecular operational taxonomic units (MOTUs) and morphospecies. Disagreements between morphospecies and MOTUs have to be explained by historically acquired geographic genetic differentiation, incomplete lineage sorting, and hybridization. The study once again highlights how important morphology still is in order to correctly interpret the results of molecular species delimitation.

## Graphical abstract

Performance of complex DNA‐based species delimitation is to be tested under simpler conditions in the absence of geographic sampling bias. Here, we present an empirical data set sampled from a single locality in a Southeast‐Asian biodiversity hotspot in Laos and investigate the performance of species delimitation approaches on a megadiverse assemblage of herbivore chafer beetles (Coleoptera: Scarabaeidae) to infer whether species delimitation suffers in the same way from exaggerate infraspecific variation despite the lack of geographic genetic variation that led to inconsistencies between entities from DNA‐based and morphology‐based species inference in previous studies.

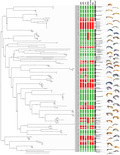

## 1 INTRODUCTION

Since DNA barcoding was formally proposed (Hebert et al., 2003, 2004; Hebert et al., 2003; Ratnasingham & Hebert, 2007), *cox1* sequences have been rapidly accumulated from all around the world (Porter & Hajibabaei, 2018). Early studies mostly had a narrow systematic focus and targeted local or regional species assemblages. With emerging global comprehensiveness from the global iBOL project (International Barcode of Life), researchers became aware of the problems that arise with the use of *cox1* (i.e., mitochondrial DNA) as taxonomic marker (Ballard & Whitlock, 2004; Dasmahapatra & Mallet, 2006; Dowton et al., 2014; Dupuis et al., 2012; Eberle et al., 2019; Funk & Omland, 2003; Nicholls et al., 2012; Ross, 2014; Smith et al., 2012), but also the effects of geographic scale on accuracy and performance of barcoding (Bergsten et al., 2012; Gaytán et al., 2020; Lohse, 2009). Therefore, geographic sampling has been a central debate (Ahrens et al., 2016; Lim et al., 2012; Reid & Carstens, 2012; Talavera et al., 2013), in particular with respect to DNA barcoding, one of the major tools of DNA taxonomy.

In order to infer in more detail the empirical behavior of species delimitation approaches that are currently in use in combination with particular genetic markers, it would be desirable to test commonly used methods on a dataset without geographic bias that still provides a sufficient number of closely related (i.e., congeneric) taxa that provide a sufficiently wide hypothesis space to test. Since low dispersal capacity might be a highly relevant factor for the outcome of such an investigation, we specifically investigate a soil‐dwelling group of beetles (Sericini chafers) which have short emergence periods, little‐specific host preferences, and a high degree of endemism (e.g., Ahrens, 2004, 2005; Dalstein et al., 2019; Eberle et al., 2017). We focus on *cox1*, since it continues and will continue to be a widely used marker for taxonomy in barcoding and metabarcoding studies.

So far, most comprehensive barcoding efforts have been made in “northern” and predominantly temperate countries (e.g., Bouchard et al., 2017; Gwiazdowski et al., 2015; Hebert et al., 2016; Hendrich et al., 2015; Pentinsaari et al., 2014; Pentinsaari et al., 2019; Rougerie et al., 2015; Rulik et al., 2017; Steinke et al., 2017; see also: http://www.ibol.org/phase1/resources/scientific‐publications/#; https://www.bolgermany.de/wp/startseite/news‐publikationen/publikationen/page/2/). The number of studies employing DNA‐based species delimitation in tropical or subtropical areas, that is, areas of high endemism and relative long‐term climatically stability, is comparatively low or limited to a narrow focal group (e.g., Ahrens et al., 2016; Astrin et al., 2012; Cancian de Araujo et al., 2019; Elias et al., 2007; Janzen & Hallwachs, 2011; Janzen et al., 2009), and only few authors assembled data on the global level (e.g., Zhou et al., 2016).

Interestingly, in regional (i.e., national)‐level libraries, molecular operational taxonomic units (MOTUs, i.e., BINs; Ratnasingham & Hebert, 2013) showed perfect matches to known morphospecies in nearly 90% of the studied species (e.g., Hendrich et al., 2015; Pentinsaari et al., 2014). Occasionally, mismatch to described species occurred due to splitting into clusters of different geographic origin (e.g., Morinière et al., 2017) or sharing of identical or closely related haplotypes among different morphospecies (e.g., Hawlitschek et al., 2017). However, matches generally decreased when geographic sampling of species was wider, for example, on a continental scale (Bergsten et al., 2012; Mutanen et al., 2016; Schmid‐Egger et al., 2019), with 12%–30% of the species resulting paraphyletic. Identification success may decrease with increasing spatial scale of sampling, up to a drop of 50% at continental scales (Bergsten et al., 2012). Sampling on a continental scale thus considerably increases the complexity of barcoding studies. Most of the “northern” latitude studies, however, are supposed to contain species with only low infraspecific haplotype diversity (due to extinctions and recolonization events during and after the Pleistocene; for example, Ahrens et al., 2013; Hewitt, 1996, 1999; Schmitt, 2007), and often assemblages only contain a small number of closely related species. Thus, these data do not represent suitable test cases of species delimitation performance when the component of actual geographic genetic variation is excluded. On the other hand, most of studies, which focused on tropical groups, collected specimens from more than one site (i.e., under geographical bias) (e.g., Elias et al., 2007; Janzen & Hallwachs, 2011; Janzen et al., 2009; Thormann et al., 2016). Often, mismatch of MOTUs with morphospecies was seen as evidence for cryptic diversity (e.g., Janzen & Hallwachs, 2011; Janzen et al., 2009).

Here we present a dataset that was sampled from one local assemblage in a Southeast‐Asian biodiversity hotspot (Laos: Phou Pan mountain). We investigate the performance of various species delimitation approaches on a megadiverse assemblage of herbivore Sericini chafer beetles (Coleoptera: Scarabaeidae). Our objective is to infer whether species delimitation suffers from exaggerate infraspecific variation in the same way that led to inconsistencies between entities from DNA‐based and morphology‐based species inference in previous studies, despite the lack of geographic genetic variation. We are interested in the degree of deep coalescence (Maddison, 1997) in this local species assemblage and in how species delimitation approaches handle these data. Excluding geographic genetic variation, we would expect less problems due to deep coalescences and thus higher rates of taxonomic congruence between morphospecies and MOTUs. Furthermore, we employ clustering algorithms similar to those used in metabarcoding approaches, to explore the reliability of this critical step in current metabarcoding analysis pipelines (e.g., Coissac et al., 2012; Deiner et al., 2017; Macher et al., 2018; Ruppert et al., 2019).

## 2 MATERIAL AND METHODS

### 2.1 Study group, sampling, and identification

The study group is the megadiverse tribe Sericini that contains worldwide nearly 4,000 described species in about 200 genera (Ahrens, 2006). They are one of the oldest lineages of phytophagous Scarabaeidae and diversified with the rise of the angiosperms 108 Ma (Ahrens et al., 2014; Eberle et al., 2017). Sericini are nearly worldwide distributed, except in Australia, most oceanic islands, archipelagos, and circumpolar regions (Ahrens, 2006). The polyphagous herbivore adults are fully winged while larvae feed on roots and underground stems of living plants (Ritcher, 1966). Some species are considered as crop pests (Nair, 1986). Their highly similar external morphology makes the species difficult to distinguish, but highly complex male genitalia are well‐differentiated between species and show only little intraspecific variation (Ahrens & Lago, 2008).

**FIGURE 1:**
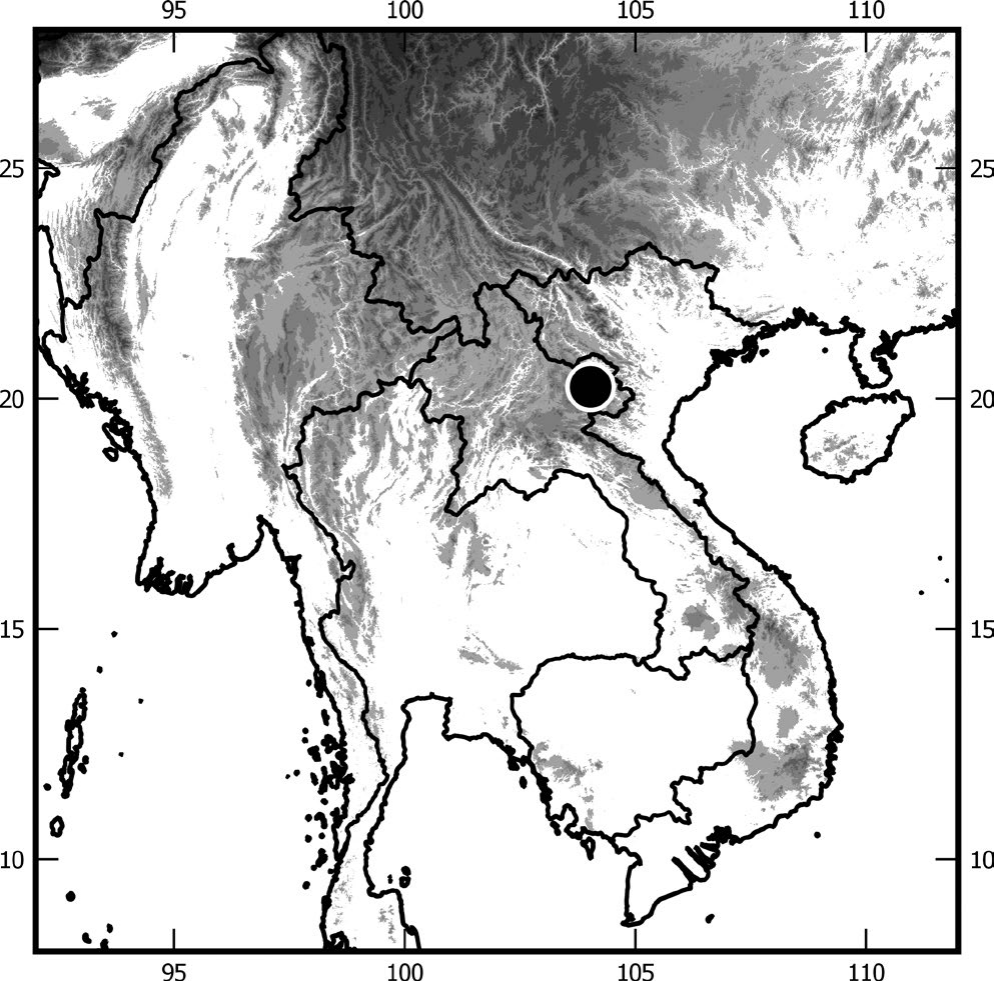
Collecting area in Laos (20°12′N, 104°01′E) (marked with a black dot)

Sampling was conducted during 4 weeks in April 2014 by Carolus Holzschuh and local collectors in the Phou Pan mountain area (Laos, Hua Phan province) (Figure 1) (ca. 20°12′N, 104°01′E), at an elevation between 1,300 and 2,000 m. Specimens were collected using light traps, by hand, or netting during daytime. The Phou Pan mountain is situated in the Indo‐Burmese biodiversity hotspot area (Myers et al., 2000) which is characterized by extremely high endemism. The habitat with its dense rainforests (Müller et al., 2013) offers a large variety of plant species for herbivore insects to feed on. For this study, we used only males (1,086 specimens), since they were assignable to distinct morphospecies, while females are often not distinguishable among closely related syntopically occurring species. Samples were pinned after DNA extraction, dry mounted, labeled, and preserved at the ZFMK (Zoologisches Forschungsmuseum Alexander König, Bonn, Germany) (see Table S1).

Specimens were presorted to morphospecies using the complex shape of their copulation organ, that is, aedeagus, which has been proven to be the best suited trait system to robustly infer species entities for this group (Dalstein et al., 2019; Eberle et al., 2016). For this purpose, male genitalia of all specimens were dissected. Habitus and genitalia of each species were photographed with a stereomicroscope (ZEISS Stereo Discovery.V20) connected to a ZEISS Axiocam. Presumably undescribed species that were not yet referable to an available species name were numbered consecutively (sp1, sp2, etc.).

### 2.2 DNA sequencing

We sequenced the *cox1* gene (5′‐end) of multiple specimens (3–5) per morphospecies (in total 190). Laboratory work followed the standard protocols of the German Barcode of Life project (Geiger et al., 2016). DNA was extracted from a mesothoracic leg and attached muscles using the Qiagen DNeasy Blood and Tissue Kit or the Qiagen BioSprint96 magnetic bead extractor.

The PCR reaction was carried out in total reaction mixes of 20 μl, including 2 μl of undiluted DNA template, 0.8 μl of each primer (10 pmol/μl), and standard amounts of the reagents provided with the “Multiplex PCR” kit from Qiagen using primers LCO1490‐JJ [5′‐CHACWAAYCATAAAGATATYGG‐3′] and HCO2198‐JJ [5′‐AWACTTCVGGRTGVCC AAARAATCA‐3′] (Astrin & Stüben, 2008). Thermal cycling was performed on Applied Biosystems 2,720 thermal cyclers (Life Technologies), using a PCR program with two cycle sets, combining a “touchdown” and a “step‐up” routine as follows: hot start Taq activation: 15 min at 95℃; first cycle set (15 repeats): 35 s denaturation at 94℃, 90 s annealing at 55℃ (−1℃ per cycle) and 90 s elongation at 72℃; second cycle set (25 repeats): 35 s denaturation at 94℃, 90 s annealing at 40℃, and 90 s elongation at 72℃; final elongation 10 min at 72℃. Unpurified PCR products were subsequently sent for bidirectional Sanger sequencing to BGI Tech Solutions.

Raw DNA sequences were assembled (forward and reverse sequence) and edited in Geneious R7 (version 7.1.3, Biomatters Ltd.) to correct base‐calling errors and to assign ambiguities (when forward and reverse sequence were not congruent for certain nucleotides). Sequences were aligned with Muscle (Edgar, 2004) as implemented into Geneious using the default settings. Primers were trimmed subsequently. All data are deposited in BOLD (project: SCOIB; https://doi.org/10.5883/DS‐DS‐SCOIBL) and GenBank (accession numbers MW128167–MW128351) respectively (see Table S1).

### 2.3 Phylogenetic analysis and species delimitation

Putative morphospecies were compared with results obtained from the DNA‐based species delimitation methods. We applied Poisson tree process (PTP) (Zhang et al., 2013), statistical parsimony network analysis (TCS) (Templeton et al., 1992), Automatic Barcode Gap Discovery (ABGD) (Puillandre et al., 2012), distance‐based clustering, and Barcode of Life database (BOLD)—Barcode Index Numbers (BINs). These methods were applied on all sequenced beetles to result in clusters that are considered molecular taxonomic units (MOTUs) (Floyd et al., 2002), that is, DNA‐based species‐assignments by the respective method.

A phylogenetic tree was calculated with maximum likelihood from the final multiple alignment of all DNA sequences using the IQ‐TREE web server (IQ‐TREE version 1.6.12; http://iqtree.cibiv.univie.ac.at/) (Nguyen et al., 2015; Trifinopoulos et al., 2016); the best substitution model (GTR+F+I+G4) was chosen with ModelFinder (Kalyaanamoorthy et al., 2017) according to Bayesian information criterion (BIC). Branch support was calculated by generating 1,000 samples for ultrafast bootstrapping (Hoang et al., 2018). The resulting tree was midpoint rooted in FigTree v1.4.3 (available from http://tree.bio.ed.ac.uk/software/figtree/). This tree was the basis for the PTP analysis. Additionally, split networks were generated using SplitsTree4 v. 4.16.1 (Huson & Bryant, 2006) to visualize incompatible and ambiguous signals in the *cox1* dataset. In these networks, the parallel edges, rather than the single branches, illustrate splits concluded from the data.

We used both versions of the Poisson tree process model (PTP) on the PTP web server (https://species.h‐its.org/; accessed on 5 August 2020): bPTP, which adds Bayesian support (pp) values to branches that delimit species in the input tree, and the refined multirate mPTP. PTP uses the shift in the number of substitutions at internal nodes to identify branching rate transition points (Zhang et al., 2013) which indicate speciation events. We used default settings for the bPTP analysis (100,000 MCMC generations, thinning: 100, burn‐in: 0.1, seed 123).

Statistical network analysis as performed with TCS v. 1.21 separates the sequence data into clusters of closely related haplotypes connected by changes that are nonhomoplastic with a 95% probability (Templeton et al., 1992); if applied to mtDNA the extent of the networks has been found to be largely congruent with morphospecies (Ahrens et al., 2007; Meier et al., 2006).

Automatic Barcode Gap Discovery (ABGD) was conducted using the ABGD web server (https://bioinfo.mnhn.fr/abi/public/abgd/abgdweb.html; accessed on 17 August 2020) with default parameters (i.e., using Jukes‐Cantor model (JC69) distances, a relative gap width of 1 and 50 steps, Pmin = 0.001, Pmax = 0.1, Nb bins for distance distribution = 20). ABGD partitions individuals for a range of prior intraspecific distances, instead of using one predefined distance threshold (Fontaneto et al., 2015; Puillandre et al., 2012). A robust result across a range of prior intraspecific distances was chosen as the best partition scheme. This outcome was also closest to the number of morphospecies and simultaneously matched the presumptive barcode gap in the histogram of distances.

Distance‐based clustering was done with the tclust‐function in the R‐package spider (v. 1.5.0; Brown et al., 2012). A threshold of 3% was applied to the pairwise distance matrix of all specimens that was corrected with the Kimura model (K80). The logic of this approach underlies most metabarcoding protocols (Liu et al., 2020; Macher et al., 2018; Piper et al., 2019), relying on the presence of a barcoding gap (Elbrecht et al., 2017), which was chosen as a gap at 3% pairwise distance by the majority of studies (however, see Beentjes et al., 2019 for an 2% example). Finally, we compared outcome from species delimitations to Barcode Index Number (BIN) assignments (Ratnasingham & Hebert, 2013) in the BOLD data base (Project—SCOIB: Sericini COI Barcoding).

**FIGURE 2:**
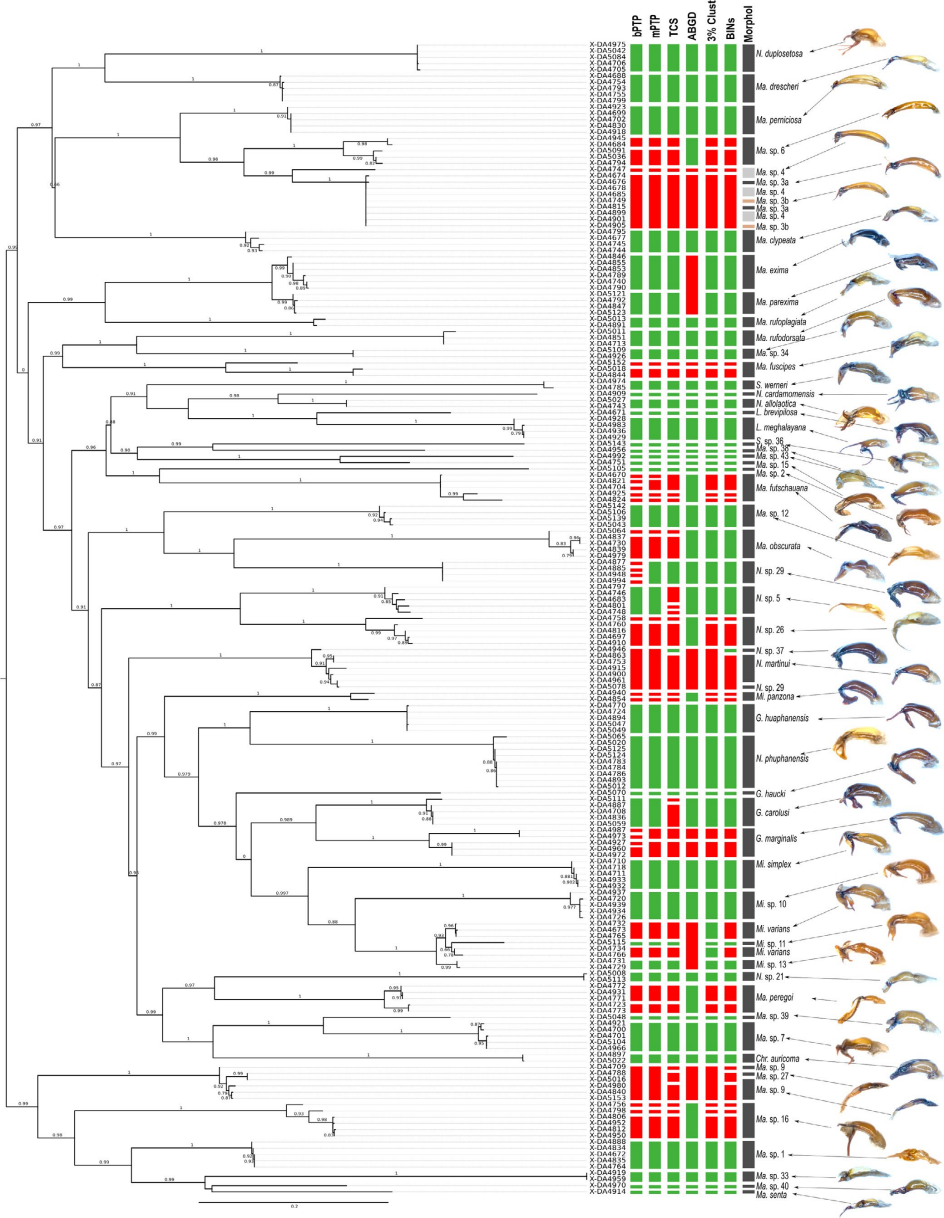
Rooted maximum likelihood tree with information about morphospecies assignments, results of species delimitations (bPTP, mPTP, TCS, ABGD, 3%‐clustering, and BOLD‐BINs) and photographs of a selection of aedeagi (lateral view). Green boxes indicate agreement between molecular species delimitation method and morphospecies assignment, while red boxes indicate disagreement. Ultrafast bootstrap supports >0.5 are shown above nodes. Genus name abbreviations: Chr.—*Chrysoserica*, G.—*Gastroserica*, Ma.—*Maladera*, Mi.—*Microserica*, N.—*Neoserica*, L.—*Lasioserica*, S.—*Serica*

To check the performance and accuracy of the DNA‐based delimitation methods compared to the a priori morphospecies hypotheses based on the genital morphology, the match ratio (Ahrens et al., 2016) was calculated: Match ratio = 2 * *N*_match_/(*N*_MOTU_ + *N*_morph_). *N*_match_ is the number of species with exact matches, when the morphospecies and DNA‐based species delimitation results to include the same specimens. N_MOTU_ is the number of classified groups by the different delimitation methods, and finally, *N*_morph_ is the number of morphospecies. All resulting MOTUs were mapped onto the phylogenetic tree beside terminal's labels (Figure 2).

## 3 RESULTS

### 3.1 Morphospecies and sequencing success

Fifty‐six morphospecies could be determined from the Phou Phan mountain area. Twenty‐seven of the morphospecies (48%) were supposedly undescribed species or could not yet be assigned to an existing species (the fauna of mainland Asia has been fully revised in terms of type specimen revision (D. Ahrens, unpublished data; D. Ahrens, personal communication, May 2020); however, several species from Indonesia are known to widely occur in the Oriental region. Some species might thus still be assigned to already described species, when taxonomic revisions are finished for all parts of Asia.

A total of 186 specimens were sequenced successfully. The length of the aligned *cox1* sequences was 658 base pairs (bp). For *Maladera* sp 16 and *Neoserica phuphanensis*, more than five individuals per morphospecies have been sequenced, since a few specimens were initially mistakenly assigned to other morphospecies. Of the 56 morphospecies 14 were singletons, that is, only represented by one specimen per species.

Due to shared haplotypes in different morphospecies, lowest inter‐ and infraspecific distances were both zero (Table 1), while maximum infraspecific distances were around 7%. The infraspecific mean distance was 1.5%, and the median even lower (0.8%). Nine morphospecies (i.e., 16% of the taxa) had infraspecific distances larger than 3%.

**TABLE 1:** Infra‐ and interspecific genetic distances of the *cox1* dataset based on morphospecies assignments, as well as number of cases beyond an arbitral 3% threshold distance being often used for MOTU clustering in Metabarcoding studies

	Interspecific		Infraspecific	
	k2p	ml	k2p	ml
Min	0	0	0	0
Max	0.24	0.24	0.074	0.073
Mean	0.17	0.18	0.015	0.015
Median	0.17	0.18	0.0083	0.0083
*N*_dist>3%_	—	—	9	9
*N*_dist<3%_	9	9	—	—

### 3.2 Species delimitation

All morphospecies included in the analysis were monophyletic with three exceptions (Figure 2): (a) *Microserica* sp 11 and *Microserica* sp 13 were nested within the clade of *Microserica varians*; (b) one of the five specimens of *Neoserica* sp 29 was within the clade of *Neoserica martinui*; and (c) *Maladera* sp 27 was placed within the clade of the morphologically very similar *Maladera* sp 9.

**FIGURE 3:**
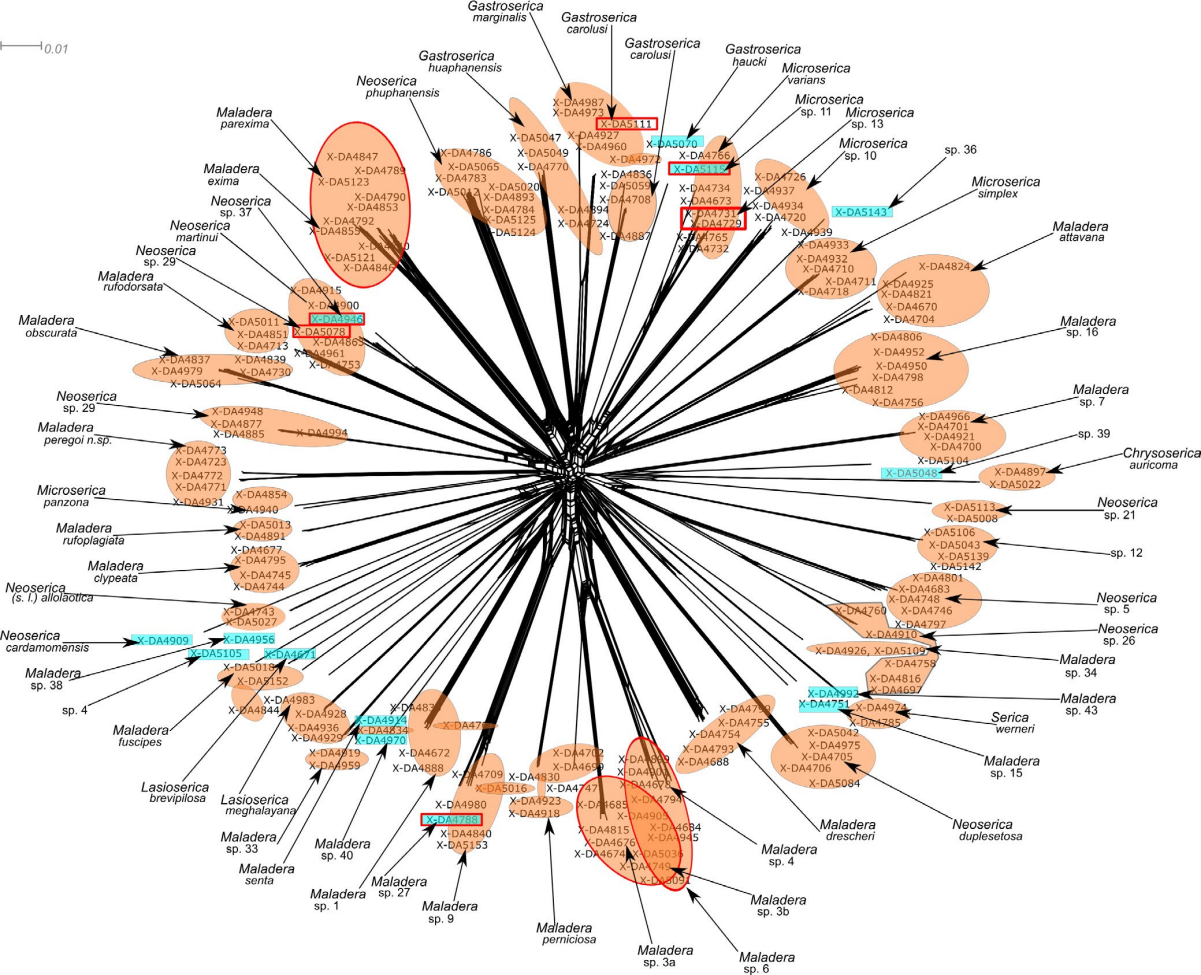
Split network of all examined specimens. Singletons are highlighted in blue squares, and others in orange colors. Morphospecies nested within others are highlighted with red squares or circles around them

Three morphospecies shared identical haplotypes (*Maladera* sp 3a, sp 3b; sp 4; Figures 2, 3). Branch support values (ultrafast bootstrapping) of morphospecies clades are high with values of 0.8 to 1. DNA‐based species delimitation applying PTP, TCS, and ABGD resulted in different clusters. Thirty‐one morphospecies showed congruent results for all DNA‐based delimitation (Figure 2). For 46 morphospecies, the results of at least one method matched with the morphospecies assignment. All methods showed splitting and also lumping of morphospecies.

bPTP and mPTP subdivided the specimens into 70 and 65 MOTUs (Table 2), with 37 (bPTP) and 38 (mPTP) matches between the morphospecies and MOTUs. Deviations are caused by erroneously inferred splitting events (i.e., individuals of one morphospecies were separated into two or more different MOTUs). Match ratios of both PTP variants were relatively low: 0.59 and 0.63, for bPTP and mPTP, respectively. TCS resulted in 69 MOTUs and had same number of matches (37) as bPTP. The match ratio (0.60) was higher than bPTP, but lower than mPTP.

**TABLE 2:** Match ratio (after Ahrens et al., 2016) of DNA‐based species delimitation methods on Sericini chafer data based on number of MOTUs and number of matches between MOTUs and morphospecies (*N*_morph_ = 56)

	bPTP	mPTP	TCS	ABGD	3%Clust	BINs
*N*_matches_	37	38	37	41	40	40
*N*_MOTU_	70	65	68	51	62	65
Match ratio	0.59	0.63	0.60	0.77	0.68	0.66

ABGD yielded 51 MOTUs and showed the highest match ratio of all delimitation methods (0.77). It was the species delimitation method that showed most lumping of different morphospecies (Figure 2). Examples for lumping are one MOTU for *Maladera exima* plus *Maladera parexima*; *Maladera* sp 9 plus *Ma*. sp 27; *Neoserica* sp 37, *Neoserica* *martinui* plus *N*. sp 29; as well as *Microserica varians*, *Mi*. sp 11 plus *Mi*. sp 13.

Distance‐based clustering at the 3% level yielded similar results to the previous methods. It found 62 MOTUs and matched with 40 morphospecies; the match ratio (0.68) was the second highest, after ABGD. Barcode Index Number (BIN) assignments revealed 65 MOTUs and matched as well with 40 morphospecies; however, its match ratio was lower (0.66) than that of 3% clustering.

In 21% of the morphospecies (*n* = 12), we found relatively deep coalescence (i.e., distinct infraspecific phylogenetic structure) (e.g., *Ma*. sp 4, sp 6, sp 16, *Ma. fuscipes*, *Ma. futschauana*, *Ma. obscurata*, *Ma. peregoi*, *N*. sp 26, *Mi. panzona*, *Mi. varians*, *G. marginalis*, *G. carolusi*). In all others, infraspecific branches were rather shallow. Taxa sampled with more than three specimens and that were represented by a single haplotype did not occur. For all those cases with deep coalescence, at least one of the DNA‐based species delimitations split the morphospecies, which in turn decreased the match ratio.

## 4 DISCUSSION

In the present paper, we investigated DNA‐based species delimitation using the mitochondrial *cox1* gene in a megadiverse assemblage of chafer beetles (Sericini) with particular focus on the performance of commonly used species delineation methods. The setup of examining barcodes of beetles from a single locality was chosen to investigate molecular species delimitation performance using data without geographic bias. While we know that match ratios strongly vary in tropical taxa (e.g., from 0.14 to 1.00; Ahrens et al., 2016), we theoretically expected that match ratios would go against one due to the exclusion of geography‐induced variance. Instead, for different standard species delimitation methods, we could also not report match ratios higher than 0.77. Interestingly, the 3% threshold clustering that is commonly used in metabarcoding approaches did not perform worse than more sophisticated approaches (like PTP or TCS); however, an accuracy of only less than 80% is not really what one could call a reliable taxonomy assessment.

DNA‐based species delimitation approaches may oversplit morphological entities (Ahrens et al., 2016), while at the same time the opposite may be also the case (Dalstein et al., 2019), even in the same taxon (as demonstrated here for the tribe Sericini). This particularly proved to be true in presence of incomplete lineage sorting and hybridization and if geographic bias is not excluded (match ratio <0.5; Dalstein et al., 2019). Extreme oversplitting has been reported for both mtDNA and nDNA, when sex‐biased dispersal occurs, which also limits general dispersal (Eberle et al., 2019).

**FIGURE 4:**
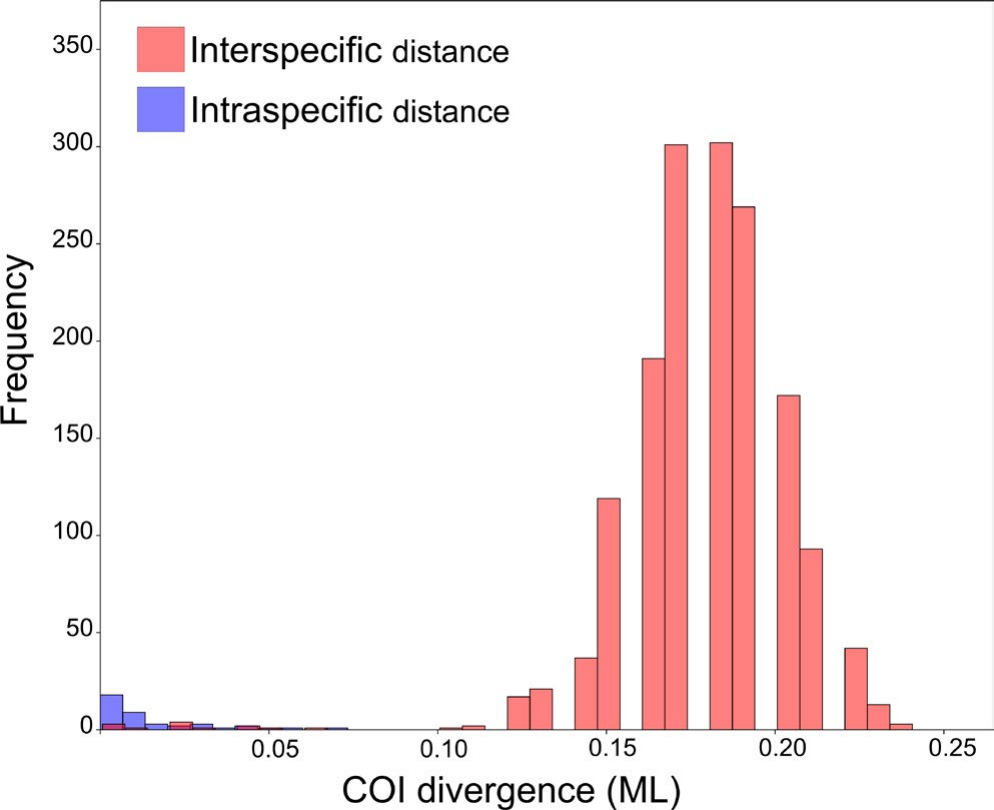
Frequency of intra‐ and interspecific distances of the Sericini data from Mt. Phou Pan (Laos)

Oversplitting in our data is caused by the relatively deep coalescence in 21% of the species, which widely corresponds with the missing match to the morphospecies (Figure 4). Also, the lack of significant interspecific divergence (i.e., a barcoding gap) seems to coincide with failure of accurate species delimitation. The impact is high with only 31 out of the 56 morphospecies matching perfectly the boundaries of inferred MOTUs (Figure 2). The nature of maternal inheritance of mtDNA and its very low recombination rate is probably the major reason for these patterns of deep coalescence. Historically acquired genetic differentiation, for example, in previously isolated populations is maintained in secondarily mixing populations (e.g., Ahrens et al., 2013). The more often such isolated populations occur in time and space, for example, due to climatic fluctuation during the Pleistocene in geographically highly structured areas such as Southeast Asia, the more often we encounter such “paleogeographically induced” infraspecific variation which leads to the same result as current geographic variation. This effect consequently impedes species delimitation methods in the same way, particularly in a single marker system (e.g., *cox1*).

In addition to such historical effects, our data also appear to have cases of hybridization and/or incomplete lineage sorting. In three cases, morphospecies were not monophyletic (*Microserica* sp 11/*Microserica* sp 13 vs. *Microserica varians*; *Neoserica* sp 29 vs. *Neoserica martinui*; *Maladera* sp 27 vs. *Maladera* sp 9.), while another three morphospecies shared identical haplotypes (*Maladera* sp 3a, sp 3b; sp 4). In all cases, we may exclude cross‐contamination based on the position of the single samples on the DNA extraction microtiter‐plates. These cases do occur in only rather closely related species, which might show similar life traits (e.g., daytime activity in *Microserica*), chemical communication, or mating behavior (which is however, unknown for all species). In those instances, lumping of morphospecies in DNA‐based species delimitation seems to be more likely; however, also oversplitting was observed (e.g., *Microserica*). Despite strong divergence in male genital morphology, hybridization between closely related species of Sericini has been reported (e.g., Dalstein et al., 2019). The rather divergent structure of the aedeagus between species might indicate a case of mechanical isolation (lock‐and‐key hypothesis) that prevents mating between different species (Eberhard, 1985). However, although there have been some recent work on the morphology of female genitalia (Özgül‐Siemund & Ahrens, 2015), our knowledge on copulation functionality and mechanics is still not sufficient to tell if morphological structures of males and female genitalia actually function as a barrier, if only through tactile recognition by cryptic female choice (Barnard et al., 2017).

Again, the present study demonstrates the necessity of an integrative taxonomy in the sense of Yeates et al. (2011) (see also Padial et al., 2010; Schlick‐Steiner et al., 2010; Tautz et al., 2002). We showed that the use of different clustering‐ and tree‐based delimitation methods (Carstens et al., 2013) with the same single maker reproduces the same erroneous signal in slightly different variations. It is thus critical to corroborate results from DNA‐based species delimitation with data from other sources (e.g., genital or larval morphology, feeding traits, behavior, etc.; e.g., Janzen et al., 2009) to allow for independent testing of species boundaries.

Sericini chafers proved to be a valuable model system, because of robust morphospecies assignments that were facilitated by highly dissimilar and morphologically complex male genitalia that serve as accurate species diagnostic trait (Dalstein et al., 2019; Eberle et al., 2016).

Overall, the initial hypothesis of impeccable DNA‐based species boundaries in syntopical species assemblages clearly had to be rejected. This was rather unexpected, especially since there was no additional evidence from other sources that these oversplittings could relate to cryptic diversity (Janzen & Hallwachs, 2011; Janzen et al., 2009, 2017).

Given the highly simplified parameters of DNA‐based species delimitation in this one‐site species assemblage, it becomes clear how complex species delimitation with DNA‐based methods is. Performance with mean error rates of more than 30% is under the expectations for proper use for applied sciences and conservation management. Methods that are more sophisticated did not perform better than over‐simplified threshold clustering methods as used, for example, in metabarcoding. Once more, we highlight the necessity of morphology for the verification of de novo species delimitation results and the constant need of integrative taxonomic approaches.

## CONFLICT OF INTEREST

We have no conflicts of interest to declare.

## AUTHOR CONTRIBUTIONS

**Daniel Lukic:** Data curation (lead); Formal analysis (lead); Writing‐original draft (lead); Writing‐review & editing (supporting). **Jonas Eberle:** Methodology (supporting); Writing‐review & editing (supporting). **Jana Thormann:** Data curation (lead); Methodology (supporting); Writing‐review & editing (supporting). **Carolus Holzschuh:** Investigation (supporting); Resources (lead); Writing‐review & editing (supporting). **Dirk Ahrens:** Conceptualization (lead); Data curation (supporting); Formal analysis (supporting); Funding acquisition (lead); Investigation (lead); Methodology (supporting); Project administration (lead); Supervision (lead); Validation (lead); Writing‐original draft (lead); Writing‐review & editing (lead).

## Supporting information

Table S1

## Data Availability

DNA sequences: Genbank accessions (MW128167–MW128351); DNA sequences (incl. abi files, locality data): BARCODE OF LIFE DATA SYSTEM (https://doi.org/10.5883/DS‐DS‐SCOIBL.%0d%0d%20).
